# Modified vaccinia Ankara expressing EEHV1A glycoprotein B elicits humoral and cell-mediated immune responses in mice

**DOI:** 10.1371/journal.pone.0265424

**Published:** 2022-03-21

**Authors:** Taylor Pursell, Jennifer L. Spencer Clinton, Jie Tan, Rongsheng Peng, Paul D. Ling

**Affiliations:** Department of Molecular Virology and Microbiology, Baylor College of Medicine, Houston, Texas, United States of America; Heinrich-Heine-University, GERMANY

## Abstract

Elephant endotheliotropic herpesvirus (EEHV) can cause lethal hemorrhagic disease (EEHV-HD) in Asian elephants and is the largest cause of death in captive juvenile Asian elephants in North America and Europe. EEHV-HD also has been documented in captive and wild elephants in their natural range countries. A safe and effective vaccine to prevent lethal EEHV infection would significantly improve conservation efforts for this endangered species. Recent studies from our laboratory suggest that EEHV morbidity and mortality are often associated with primary infection. Therefore, we aim to generate a vaccine, particularly for EEHV1 naïve animals, with the goal of preventing lethal EEHV-HD. To address this goal, we generated a Modified Vaccinia Ankara (MVA) recombinant virus expressing a truncated form of glycoprotein B (gBΔfur731) from EEHV1A, the strain associated with the majority of lethal EEHV cases. Vaccination of CD-1 mice with this recombinant virus induced robust antibody and polyfunctional T cell responses significantly above mice inoculated with wild-type MVA. Although the vaccine-induced T cell response was mainly observed in CD8^+^ T cell populations, the CD4^+^ T cell response was also polyfunctional. No adverse responses to vaccination were observed. Overall, our data demonstrates that MVA-gBΔfur731 stimulates robust humoral and cell-mediated responses, supporting its potential translation for use in elephants.

## Introduction

Elephant endotheliotropic herpesvirus (EEHV) can cause lethal hemorrhagic disease (EEHV-HD) primarily, but not exclusively in juvenile elephants, both in captivity and in the wild [1]. Mortality from EEHV infection has been observed most often in Asian elephants, as opposed to African elephants, and is largely caused by two chimeric variants, EEHV1A and EEHV1B [1, 2]. While both EEHV4 and EEHV5 are also endemic in Asian elephants and can cause significant morbidity, documented fatal cases have been rare [3, 4]. EEHV types 2, 3, 6, and 7 are recognized as endemic in African elephants [1]. Recently, morbidity and mortality caused by EEHV3 infection in several African elephants has been observed, raising concern over EEHV-HD in African elephant populations as well [5, 6]. Among Asian elephants, juveniles between the ages of 2 and 8 years appear to be most vulnerable to EEHV-HD [1]. While there are treatment plans available for EEHV disease, the rapid onset of viremia makes it difficult to diagnose EEHV and start treatments in a timely manner. In addition, there are no licensed therapeutics or vaccines available that are known to prevent EEHV-HD in elephants. Therefore, we sought to develop a vaccine that elicits immunity sufficient to prevent lethal EEHV-HD.

Accumulating evidence suggests that both humoral and cellular adaptive immune responses are important for controlling and clearing herpesvirus infections, and will be required for an effective vaccine to prevent EEHV-HD [7–15]. We have recently shown that decline of either maternally-transferred anti-EEHV antibodies or absence of anti-EEHV antibody levels, especially those specific for the EEHV type causing illness, correlate with EEHV-HD susceptibility in calves [16]. These results also suggest that morbidity and mortality from EEHV is caused from a primary infection rather than reactivation. Additionally, cell-mediated immunity (CMI) carried out by T cells also plays an important role in controlling herpesvirus reactivation events [17]. Inadequate CMI and T cell responses have been described as a risk factor for developing primary human cytomegalovirus (hCMV) [18–22], varicella-zoster virus (VSV) [23, 24], Epstein-Barr virus (EBV) [25], and human herpesvirus type 6 (HHV-6) [26] infections and/or undergoing reactivation events in humans. Based on the apparent absence of detectable strain-specific anti-EEHV antibodies prior to development of EEHV-HD, and essential CMI responses required for human herpesvirus infections, we hypothesize that inducing both potent antibody responses and CMI will be critical for developing a successful EEHV vaccine.

In this study, we investigated the immunogenicity of a recombinant Modified Vaccinia Ankara (MVA) expressing an EEHV1A glycoprotein B (gB) to induce humoral and cellular immune responses in mice [27, 28]. The MVA system was chosen for several reasons: (1) several poxviral vectored vaccines have been licensed for veterinary pathogens, (2) MVA has an extensive safety record and ability to produce robust T cell responses against glycoprotein vaccine targets in a variety of model systems [27–30], and (3) MVA has been used as a vaccine against cowpox in Asian elephant herds in Europe with no known side effects [31]. Additionally, glycoproteins have been exploited as vaccine targets for many viral diseases due to their fundamental role in cell entry. They are also suitable vaccine targets based on their ability to induce robust neutralizing antibody titers and strong, durable T cell responses [29]. Specifically, multiple herpesvirus glycoprotein subunit vaccines are being evaluated or are licensed for humans [29, 32–34]. In EEHV, gB is relatively well-conserved across EEHV species and we have observed antibody cross reactivity between all EEHV species endemic in Asian elephants, suggesting the presence of well-conserved B-cell epitopes [16]. In addition to inducing robust antibody responses, we also observed that gB was the most immunoprevalent target for CMI in a majority of chronically infected adult elephants [35].

Here, we describe the construction, characterization, safety, and immunogenicity of the first candidate EEHV vaccine utilizing MVA to express EEHV1A gB. Overall, our data demonstrates that an MVA engineered to express the extracellular domain of EEHV1A gB induces robust humoral and CMI responses, making it a strong vaccine candidate for the prevention of lethal EEHV-HD infection.

## Materials and methods

The animal work described in this manuscript was approved by the Baylor College of Medicine IACUC under protocol AN-7938. Vaccinated mice were euthanized according to the IACUC-approved Euthanasia in Rodents Policy and the CCM Policy, which includes Carbon Dioxide Gas (CO2) + a secondary method (bilateral opening of the thorax).

### Cells, viruses, plasmids, and animals

UMNSAH/DF-1 (CRL-12203) and 293T (CRL-3216) cells were purchased from the American Type Culture Collection. The cells were maintained in DMEM supplemented with 10% FBS at 39°C for the DF-1 Cells and 37°C for the 293T cells. Umbilical cord elephant endothelial cells (EEC) were isolated as described previously [36] and maintained at 37°C in EBM-2 media (Lonza) supplemented with 10% FBS. EEC1 and EEC2 cells were isolated from umbilical cords of one male and one female elephant born at the Houston zoo respectively. EEC3 cells are immortalized cells generated by transduction of EEC2 cells with a lentivirus expressing the SV40 Large T antigen. Wild-type (wt) MVA was purchased from the American Type Culture Collection (VR-1508). The shuttle vector pLW-73 was kindly provided by Linda Wyatt and Bernard Moss at the National Institutes of Health (NIH). Mice used in this study were outbred 6 to 8-week-old female CD-1 mice and were purchased from the Center for Comparative Medicine at Baylor College of Medicine.

### Generation of EEHV1A MVA-gBΔfur731

**Fig 1:**
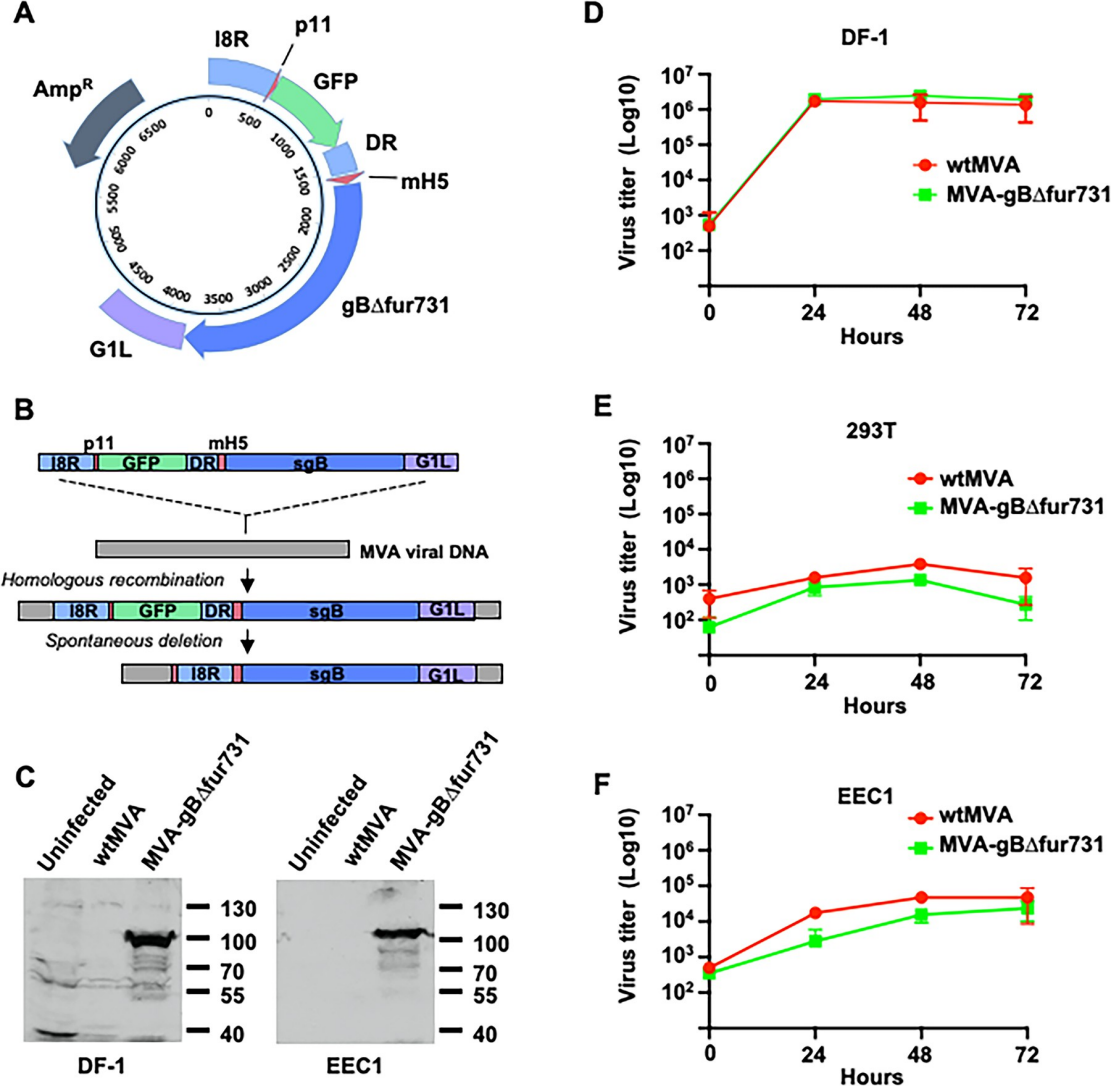
Construction of an MVA-gBΔfur731 recombinant and its replication in selected cell lines. (**A**) Shuttle vector plasmid map of pLW-73 containing gB coding regions (gBΔfur731), MVA sequences homologous to the MVA I8R and G1L genes for homologous recombination, a GFP reporter, a direct repeat sequence (DR) homologous to the I8R gene to facilitate spontaneous deletion of the GFP gene, and ampicillin resistance gene. (**B**) Schematic overview of recombination events leading to generation of final MVA-gBΔfur731 virus recombinants, including homologous recombination and spontaneous deletion of GFP. (**C**) Expression of gBΔfur731 by SDS-PAGE and Western blotting of infected DF-1 and EEC1 cell lysates. gB was detected by antisera from a chronically EEHV-infected elephant. (**D**) Replication of wtMVA and MVA-gBΔfur731 in DF-1 cells. Cells were infected with an MOI of 0.05 and cells and supernatants were harvested and combined at 0, 24, 48, and 72 hours post-infection. Virus yields were determined by MVA immunostaining assay in DF-1 cells. The titers represent the average of two independent experiments with error bars representing standard deviation. (**E**) Same as D, except virus infection in 293T cells. (**F**) same as in D, except virus infection in EEC1 cells.

A recombinant MVA expressing gB was generated using previously described methods [37, 38]. Briefly, the EEHV1A Kimba U39 (gB) sequence [39] encoding amino acid residues 1–731 lacking the transmembrane and cytoplasmic domains (amino acid residues 732–850) and the furin cleavage site (amino acid residues 433–436) was codon optimized, synthesized, and cloned into the pLW-73 shuttle vector [38, 40, 41]. The final gB cDNA encodes a protein of 727 amino acid residues and is designated gBΔfur731 to indicate deletion of the predicted furin cleavage site and the last amino acid residue position prior to the deletion of transmembrane encoding sequences. Protocols for generating MVA recombinants with pLW-73 and description of major features of this shuttle vector have been described in detail elsewhere [37, 38, 40, 41]. Briefly, important features of the pLW-73 vector include: (1) MVA sequences for I8R and G1L that enable insertion of foreign genes between these open reading frames, (2) a GFP gene under the control of the p11 promoter, (3) a direct repeat sequence for the 3’ end of the I8R gene downstream of the GFP gene to facilitate homologous recombination and spontaneous deletion of the GFP gene, and (4) an m15 promoter for driving expression of foreign genes. Because 18R and G1L are essential for MVA viability, insertion of foreign genes between these loci is predicted to select against variants with a deletion of the inserted gene [38, 41]. A diagrammatic representation of these features can be found in Fig 1A.

Recombinant shuttle plasmid DNA was transfected into DF-1 cells using JetPRIME transfection reagents (Polyplus) according to the manufacturer’s instructions. Media was removed from transfected cells, and cells were infected with wtMVA for 90 minutes at 39°C. After infection, media was replaced, and cells were cultured at 39°C for 1–2 days. Infected cells were collected and freeze-thawed three times before infecting fresh DF-1 cells. Fluorescent-activated cell sorting (FACS) was used to isolate GFP-positive cells containing MVA recombinants in single cell suspensions, which were sorted into 50 μL water per well in 96-well plates. Two hundred μL of 5x10^4^ DF-1 indicator cells were plated into each well and incubated at 39°C for 1–2 days. GFP-positive wells were harvested by manual disruption of cells. Some cells were reserved for preparing DNA for PCR analysis, while the rest was frozen and thawed three times to prepare a virus stock for further purification and amplification. Recombinant MVAs were confirmed by PCR using primers 5’-GTGTCCTGAGTATAGCCCAAAG-3’ and 3’-GGAAAGAAGAACTCTAGGGTGT-5’, and the following cycle conditions: 98°C for 2 minutes, 29 cycles of 95°C for 15 seconds, 52°C for 30 seconds, and 68°C for 1 minute, 68°C for 4 minutes, and 10°C hold. Virus stocks from wells confirmed by PCR to contain gBΔfur731 sequences were seeded on DF-1 cells and subjected to two rounds of plaque purification. Following the second round, GFP-negative plaques, presumably generated by spontaneous deletion of the GFP gene facilitated by the DR sequences homologous to the flanking I8R gene were picked, amplified and plaque purified again. Final amplified virus was analyzed by PCR and PCR sequencing to determine that no wtMVA was present and to confirm that no mutations or deletions were introduced during the virus purification process.

### Production and purification of MVA-gBΔfur731

Large scale virus amplification and purification was performed as described previously [38, 40]. Briefly, six 15-cm^2^ culture dishes containing approximately 6.6x10^6^ DF-1 cells per plate were infected with the final MVA-gBΔfur731 recombinant virus at a multiplicity of infection (MOI) of 0.05. After 5–7 days, cells were harvested with cell scrapers, and cell supernatants and pellets were centrifuged at 670 ×g for 10 minutes at 4°C. Supernatants were transferred to new bottles and kept separately. Pelleted DF-1 cells were resuspended in 2 mL of 10μM Tris buffer pH 9.0, freeze/thawed three times, sonicated at 60 Hz for 1 minute, and centrifuged at 670 ×g for 5 minutes. Supernatants were removed and combined with the original tissue culture supernatants. Supernatant mixtures were centrifuged at ~30,000 ×g for 55 minutes at 4°C prior to discarding final supernatants. Pellets containing virus were resuspended in 10μM Tris buffer pH 9.0 and added to a 36% sucrose/10μM Tris buffer pH 9.0 cushion and centrifuged at ~30,000 ×g for 2 hours. Leftover liquid was aspirated and MVA-gBΔfur731 viral pellets were resuspended in sterile saline (0.9% Sodium Chloride), aliquoted, and frozen at -80°C.

### MVA immunostaining assay

Recombinant MVA-gBΔfur731 and wtMVA virus titers were performed on DF-1 cells. DF-1 cells were seeded at 4x10^5^ cells/well in 24-well plates, infected with 100μL of 10-fold serial dilutions of virus stock for 2 hours, overlaid with growth media containing 0.5% methylcellulose, and incubated for 2 days at 39°C. After incubation, cells were washed with PBS, fixed with 1:1 methanol:acetone, and blocked with PBS/4% FBS for 1 hour. Rabbit anti-vaccinia antibody (Invitrogen, PA1-7258) was diluted in PBS/4% FBS (1:500) and incubated with cells for 1.5 hours at room temperature (RT). After washing with PBS and PBS/4% FBS, goat anti-rabbit horseradish peroxidase conjugated antibody (Jackson ImmunoResearch Laboratories Inc., 111-035-144) was diluted in PBS/4% FBS (1:500) and incubated with cells for 45 minutes at RT. ImmPACT DAB Peroxidase Substrate (Vector Labs, SK-4105) was diluted in appropriate kit buffer and developer was added to cells for 10 minutes. Plates were washed twice with H_2_O, dried overnight, and plaque spots counted for quantification.

### Immunoblot assay

MVA infected DF1 or EEC1 cells were mixed with 2x sample loading buffer, resolved by SDS-PAGE and immunoblots carried out using elephant serum and anti-elephant IgG as described previously [42].

### Virus replication

For analysis of virus replication and spread, DF-1, 293T, and EECs were infected with an MOI of 0.05. Cells and supernatant were harvested at 0, 24, 48, and 72 hours post-infection, freeze-thawed three times and samples assayed by immunostaining assay. Results are from two independent infection experiments and plaque assays were performed in duplicate for each experiment.

### Mouse immunizations

Mice were separated into treated or control groups (*n* = 6–15). The virus dose and vaccination schedule used are identical to multiple similar studies done previously [43–46]. Treated groups received 100 μL of 1x10^7^ PFU MVA-gBΔfur731 in sterile saline, while control groups received 100 μL of 1x10^7^ PFU wtMVA in sterile saline. Mice received up to three intraperitoneal injections at four-week intervals, yielding vaccinated prime, prime-boost, and prime-two boost groups. Mice were euthanized four weeks after each vaccination, serum was harvested for the Luciferase Immunoprecipitations System (LIPS) assays, and spleens were processed for cellular staining and flow cytometric analysis. All procedures used in this study were approved by the Baylor College of Medicine Institutional Animal Care and Use Committee.

### Immunologic assays for evaluation of gB-specific immune responses

#### gB-specific LIPS assay

Preparation of GLuc-antigen fusion constructs were performed as previously described [16, 47]. Briefly, cDNAs for EEHV1A gB and gH lacking transmembrane and cytoplasmic domains, were codon optimized, synthesized (GenScript) and subcloned into the pGaus3 expression plasmid [48, 49]. Expression plasmids were transfected into HEK 293T cells, and supernatants and cell extracts were harvested and stored at -80°C. EEHV1A gB/U39 is identical to the cDNA sequence used for generating the MVA recombinant virus and the gH protein was derived from EEHV1A Kimba sequences [39]. LIPS analysis was performed as previously described [16, 50]. Briefly, serum samples were diluted 1:10 in Buffer A (50mM Tris pH 7.5, 100mM NaCl, 5mM Mg Cl2, 1% Triton X-100) for storage up to a month at 4°C. For use in the assay, samples were diluted 1:5 in the same buffer. Luminescence units (LU) of stored GLuc extracts were determined on the day of the assay and a mastermix of each GLuc extract containing 1x10^7^ LU per 50 μL was made. Fifty μL of GLuc extract mastermix was added to 50 μL of diluted serum (in duplicate) and incubated on a shaker at RT for 1 hour. After 1 hour, GLuc extract-diluted serum was added to 5 μL of a 30% Protein A-G bead suspension (diluted in PBS) in a 96-well filter plate (Millipore). Samples were incubated with the beads for one hour at RT with constant shaking. Beads were then washed five times with Buffer A followed by two times with PBS using a 96-well vacuum manifold (Millipore). An OmniStar automatic plate luminometer (BMG Labtech) was used to inject 50 μL of Renilla luciferase assay substrate into well, shake for 2 seconds, and record relative light unit (RLU) values for 5 seconds. Values reported represent the average RLUs over 5 seconds post-injection.

#### gB-specific T cell responses

Mouse spleens were processed by trimming excess fat, cutting spleens into multiple pieces, and mashing spleen remnants though a 40μm filter. Red blood cells were lysed and splenocytes were centrifuged at 350×g for 5 minutes before resuspending in complete RPMI-1640 media supplemented with 10% FBS, penicillin/streptomycin, and 2-mercaptaethanol. Cells were pelleted at 350×g for 5 minutes, resuspended in 1mL of 90% FBS/10% DMSO per spleen, and frozen until analysis. When analyzed, cells were thawed in a 37°C water bath and added to pre-warmed complete RPMI-1640 media. Splenocytes were centrifuged at 350×g for 5 minutes and washed once with pre-warmed RPMI-1640 media. After cells were revived, 2x10^6^ splenocytes were plated on 96-well round bottom plates and were stimulated at 37°C overnight with EEHV1A gB peptide pools at 1ug/mL in complete RPMI-1640 medium. The EEHV1A glycoprotein B peptides were each synthesized and purified by GeneWiz at 1 mg per vial. Peptide libraries are consecutive 15-mers overlapping by 11 amino acids spanning the length of gB (210 peptides). Three peptide pools (denoted gB1, gB2, and gB3) were made containing 70 individual peptides per pool and reconstituted in DMSO at a concentration of 10 mg/mL [35]. After peptide stimulation, Brefeldin A (BD Bioscience) and Monensin (2 mg/mL) were added and the cells were incubated an additional 6 hours at 37°C. At the end of the stimulation period, cells were washed and incubated with Fc Block (BD Biosciences) for 15 minutes at 4°C. Cells were stained with surface marker antibodies diluted in staining buffer (PBS, 5%FBS, 0.05% sodium azide) at 4°C for 30 minutes in the dark. Cells were subsequently washed in staining buffer, and fixed in Cytofix/Cytoperm (BD Biosciences) for 20 minutes at 4°C. After fixation, splenocytes were permeabilized with 1× perm/wash solution (BD Biosciences) before intracellular cytokine staining. Cells were stained with intracellular cytokine antibodies diluted in a 1:1 mixture of Brilliant Stain Buffer (BD Biosciences) and 2× perm/wash buffer at 4°C for 30 minutes in the dark. Stimulated spleen cells were washed in perm/wash buffer and resuspended in analysis buffer (PBS without Mg^2+^ and Ca^2+^, 2% FBS, 2mM EDTA, 25mM HEPES) prior to flow cytometric analysis. Dead cells were excluded using GhostRed780 kit (Tonbo). The fluorochrome-conjugated, anti-mouse antibodies used are described in Table 1. Cells were analyzed using an LRSII (BD Biosciences) and data analysis was performed using the FlowJo software version 10.7 (BD Biosciences). The number of CD3^+^ events ranged between 1x10^5^ and 2x10^5^. Background responses in negative controls were subtracted from stimulated samples.

**Table 1:** Specific antibodies used to assess CMI in vaccinated mice.

Antibody Target	Fluorophore	Dilution	Manufacturer	Catalog #	Clone
CD3e	PerCP-Cy5.5	1:25	Tonbo	65-0031-U100	145-2C11
CD4	BUV395	1:400	BD Biosciences	563790	GK1.5
CD8a	FITC	1:800	Tonbo	35-0081-U500	53–6.7
IFNγ	BV421	1:50	BD Biosciences	563376	XMG1.2
IL-2	APC	1:200	Tonbo	20-7021-U100	JES6-5H4
TNFα	PE/Dazzle 594	1:50	Biolegend	506346	MP6-XT22
Viability	GhoseDye780	1:50	Tonbo	13-0865-T500	

#### Statistical analysis

Statistical analysis was done with GraphPad Prism software version 8.4.3 (San Diego). For LIPS results for different groups are presented as geometric means ± the standard deviation (SD on log_10_ values. Normality of the data sets were determined using the Shapiro-Wilk test. Significance of normally distributed data were determined by one-way ANOVA with Tukey’s multiple comparisons test with a *p* value < 0.05 denoting significant differences. The cut-off level for determining sensitivity and specificity for each viral antigen was derived from the mean antibody titer of the uninfected samples plus 5 SD. For flow cytometry assays, results are presented as median ± the standard deviation (SD) of percent of positive cells. Normality of the data sets were determined using the Shapiro-Wilk test. Significance of normally distributed data were determined by two-way ANOVA with Tukey’s multiple comparisons test with a *p* value < 0.05 denoting significance. Comparisons between empty vector and MVA-gBΔfur731 vaccinated groups were done using the Mann-Whitney U test with a significance set at a *p* value < 0.05. Outliers were identified by ROUT analysis and removed.

## Results

### Generation and characterization of MVA gBΔfur731

Previous attempts to express full-length EEHV1 gB in MVA were unsuccessful, indicating potential toxicity of the full-length protein. In contrast, an hCMV gB protein lacking its transmembrane and cytoplasmic domains has been successfully expressed in a recombinant MVA and is progressing through clinical trials as a vaccine against hCMV [51, 52]. This truncated version of hCMV gB has also been demonstrated to be significantly more immunogenic in than full-length gB [53, 54]. Thus, a 727 amino acid residue EEHV1A gB sequence lacking the transmembrane domain and internal furin cleavage site was codon-optimized, synthesized, and cloned into the pLW-73 shuttle vector (pLW-73 gBΔfur731) [38, 41] (Fig 1A). We have initially selected the EEHV1A sequence for gB because the majority of morbidity and mortality is associated with this variant of EEHV1. Homologous recombination with wtMVA was induced in DF-1 chicken cells as described previously [38, 40] (Fig 1B). Successful MVA and shuttle vector recombination yielded GFP-positive virally-infected cells that were isolated by FACS [40]. A recombinant virus was plaque purified two times based on expression of GFP. Subsequently, a GFP-negative virus, which spontaneously deleted the GFP gene via homologous recombination between the DR and the upstream I8R gene, was subjected to two rounds of plaque purification to yield a virus designated as MVA-gBΔfur731 (Fig 1B).

Purified stocks of the MVA-gBΔfur731 virus were prepared as described previously [38, 40] and analyzed by targeted sequencing of the insertion and for expression of gBΔfur731. Sequencing confirmed that no changes were observed in the gBΔfur731 gene or within a few hundred bases on either side of the insertion site in MVA virus genome. Multiple virus stocks were generated by nine successive virus passages (P9) and sequencing of the gBΔfur731 insertion indicated no mutations or deletions within the gene or surrounding sequences, indicating that the recombinant virus stably maintained the gBΔfur731 gene. To ensure adequate expression of gBΔfur731 from recombinant MVA, DF-1 cells and elephant primary endothelial cells were infected with MVA-gBΔfur731 or wtMVA. Immunoblotting using sera from an adult elephant with prior EEHV1 infection [55, 56] and known gB immunoreactivity [16] detected a protein with a molecular weight expected for gBΔfur731 in both chicken and elephant cells (Fig 1C). The predicted molecular weight of native unmodified gBΔfur731 is 83.3kDa. However, it is expected that one or more post-translational modifications of gBΔfur731 would occur, including addition of carbohydrates, which is consistent with detection of a protein migrating at approximately 100kDa. Despite deletion of the transmembrane domain, no gBΔfur731 was detected in supernatants of infected cells. Importantly, MVA does not replicate in most mammalian cells [57, 58]. To confirm that insertion and expression of gBΔfur731 did not enhance predicted growth characteristics of the virus, we tested its ability to replicate in chicken, human, and elephant cells. As expected, MVA-gBΔfur731 replicated to significantly high titers in DF-1 chicken cells, while no appreciable replication was observed in either 293T human cells, primary EECs (EECs 1 and 2), or immortalized EECs (EEC3) (Fig 1D–1F and Table 2).

**Table 2:** Replication of MVA and MVA-gBΔfur731 after Low Multiplicity Infections^a^.

					Virus Replication^b^	
Cell Line	ATCC code	Species	Organ	Morphology	wtMVA	MVA-gBΔfur731
DF1	CRL-12203	Chicken	Embryo	Fibroblast	70 (P)	90 (P)
293	CRL-1573	Human	Kidney	Epithelial	0.01 (NP)	0.02 (NP)
EEC1	NA	Elephant	Umbilical cord	Endothelial	0.95 (SP/NP)	0.46 (NP)
EEC2	NA	Elephant	Umbilical cord	Endothelial	ND	0.05 (NP)
EEC3	NA	Elephant	Umbilical cord	Endothelial	ND	0.5 (NP)

^a^ MOI = 0.05.

^b^ Virus yield at 72 hours was divided by the input titer to determine virus replication (increase in virus titer). Permissive (P); >25-fold increase, Semi-permissive (SP); 1 to 25 fold increase, and Nonpermissive (NP); <1 fold increase. Permissive replication statuses are indicated in the parentheses.

### MVA-gBΔfur731 vaccination induces robust gB-specific antibody responses

**Fig 2:**
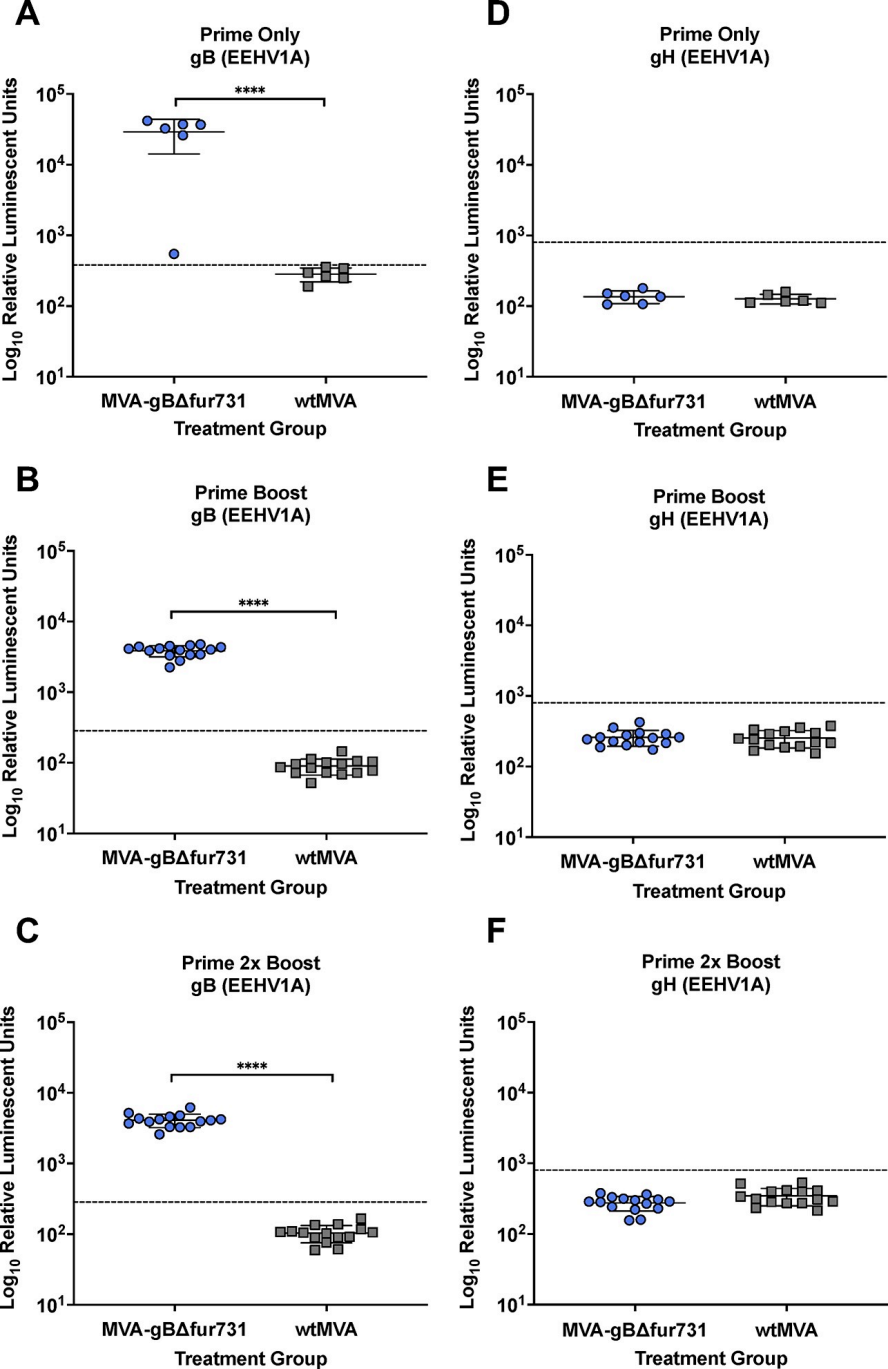
MVA-gBΔfur731 induces anti-gB antibody responses in mice. Anti-gB-specific antibody titers in vaccine trial groups: (**A**) prime vaccine only, (**B**) prime and boost vaccines, (**C**), prime with 2 boost vaccines. Anti-gH-specific antibody titers in vaccine trial groups: (**D**) prime vaccine only, (**E**) prime and boost vaccines, (**F**), prime with 2 boost vaccines. Serum samples from MVA-gBΔfur731 or wtMVA inoculated mice are represented by blue circles and gray squares respectively. Antibody levels are expressed in RLUs and plotted on a log_10_ scale. Mean ± SD values for each are shown, with each symbol representing the mean result for one animal with two replicates. Asterisks (****) indicate the statistically significant difference (*p*<0.0001) between MVA-gBΔfur731 and MVA immunized groups, as determined by the one-way ANOVA with Tukey’s multiple comparisons test. The dashed line indicates the cut-off level for determining the sensitivity and specificity for each viral antigen, and is derived from the mean antibody titer of seronegative serum samples or no serum controls plus 5 SD.

Outbred CD-1 mice were used to study the safety and immunogenicity of the MVA candidate vaccine expressing EEHV1A gBΔfur731. CD-1 outbred mice were chosen for this study with the hypothesis that outbred small-animal models might exhibit a range of host immune responses induced by vaccination and possibly mimic the range that might be seen in outbred elephants, the eventual species targeted for vaccination. An overview of the vaccination schedule and study design is presented in Table 3. Mouse sera were harvested at 4 weeks following the prime vaccination and after each of the two boosts. Antibody responses following vaccination were evaluated using the LIPS assay as previously described [16, 47]. Animals in the prime only group that received the MVA-gBΔfur731 vaccination produced significantly higher titers of gB-specific antibodies (~2 logs) than animals that received the wtMVA vaccine (Fig 2A). Additionally, mice that received either one or two boost vaccinations of MVA-gBΔfur731 induced significantly higher gB antibody levels than those that received two wtMVA injections, although there was no difference between groups that received either a single or two-booster vaccinations (Fig 2B and 2C). No antibody responses were detected in any of the vaccinated groups to an unrelated EEHV glycoprotein, gH (Fig 2D–2F). Importantly, there were no signs of illness or side-effects seen in immunized mice throughout the study duration. Thus, these results indicate that MVA-gBΔfur731 vaccinated mice elicit robust gB-specific antibody responses.

**Table 3:** Overview of vaccination groups and immunization schedules.

Group	Mice (*n*)	Treatment 1 (Day 0)	Treatment 2 (4 weeks)	Treatment 3 (8 weeks)	Sample time point and type
Prime only	6	wtMVA			4 weeks, Serum
	6	MVA-gBΔfur731			
Prime-boost	15	wtMVA	wtMVA		8 weeks, Serum
	15	MVA-gBΔfur731	MVA-gBΔfur731		
Prime-2 boosts	15	wtMVA	wtMVA	wtMVA	12 weeks, Serum and splenocytes
	15	MVA-gBΔfur731	MVA-gBΔfur731	MVA-gBΔfur731	

### MVA-gBΔfur731 vaccination induces a strong T cell response

**Fig 3:**
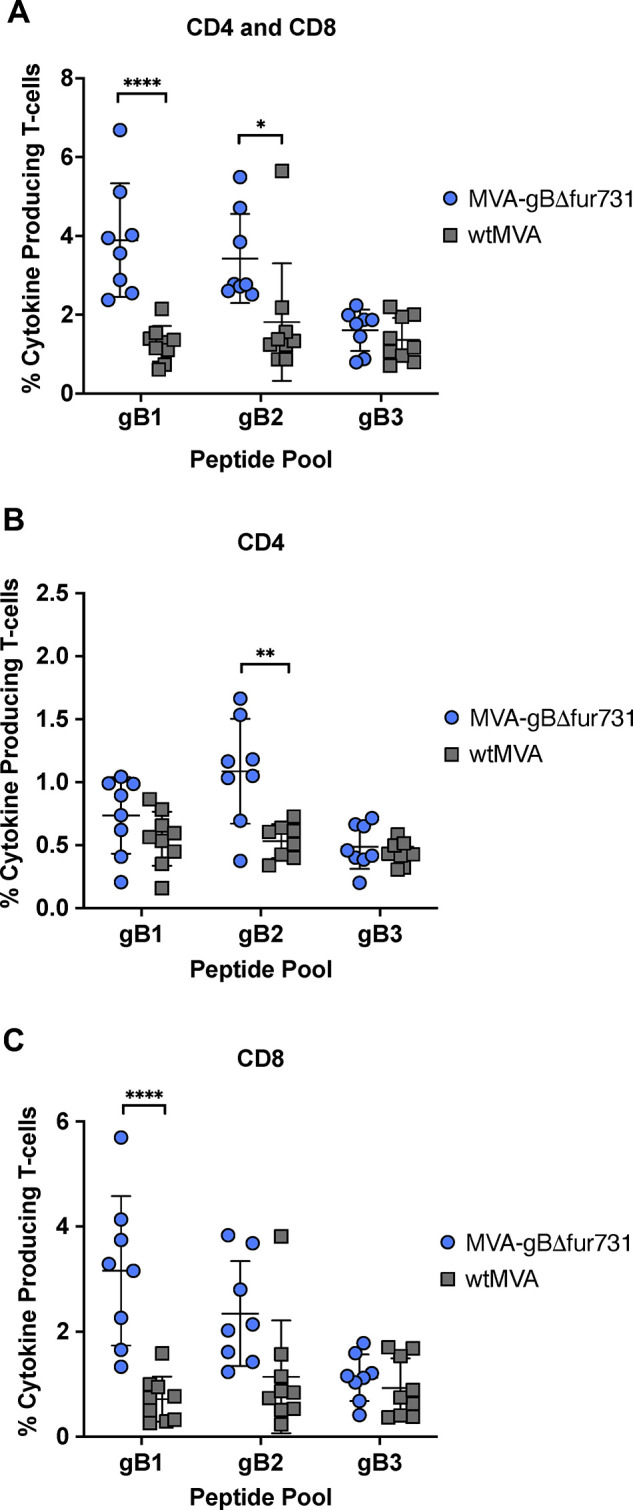
Induction of gB-specific T cell responses after immunization. Splenic T cells from immunized mice were assessed for cytokine production after stimulation with gB peptide pools. Blue circles represent splenic T cells from animals immunized with MVA-gBΔfur731 and gray squares represent mice immunized with wtMVA. (**A**) Percentage of CD4 and CD8 cells producing at least one of three cytokines IFNγ, TNFα, or IL-2. (**B**) Percentage of CD4 cells producing at least one of three cytokines IFNγ, TNFα, or IL-2. (**C**) Percentage of CD8 cells producing at least one of three cytokines IFNγ, TNFα, or IL-2. Median values ± SD values for each group are shown as horizontal lines and error bars. Asterisks (*/**/****) indicate statistically significant differences (*p*<0.05/*p*<0.01/*p*<0.0001) between stimulation groups or MVA-gBΔfur731 and MVA immunized groups, as determined by two-way ANOVA with Tukey’s multiple comparisons test. A *p* value < 0.05 denotes significance.

Splenocytes harvested from MVA-gBΔfur731 or wtMVA vaccinated mice from the prime-2 boosts group were used to assess CMI responses. Mouse splenocytes were stimulated with three gB-specific peptide pools representing the entire gB protein [35] and analyzed by flow cytometry to evaluate cytokine production in T cells. Live T cells were gated based on viability dye, CD3^+^, and CD4^+^ or CD8^+^ extracellular markers, and stained for intracellular cytokines. Intracellular cytokines indicative of activated T cell responses included interferon-gamma (IFNγ), tumor necrosis factor-alpha (TNFα), or interleukin-2 (IL-2). Total cytokine producing T cells (CD4^+^ and CD8^+^), defined as T cells of either type that express at least one of the intracellular cytokines above, were significantly higher in mice receiving the MVA-gBΔfur731 vaccine and stimulated with gB1 or gB2 peptide pools (Fig 3A). Additionally, splenocytes stimulated with gB2 peptides after vaccination with MVA-gBΔfur731 had significantly higher percentages of cytokine producing CD4^+^ cells (Fig 3B). Lastly, gB1 stimulated splenocytes from mice that received MVA-gBΔfur731 vaccines had significantly increased percentages of cytokine producing CD8^+^ T cells (Fig 3C). While those splenocytes treated with gB2 peptides had an increasing trend in cytokine producing CD8^+^ T cells, these data were not statistically significant. Overall, more CD8^+^ T cells (up to 6%) exhibited higher levels of cytokine production than that of CD4^+^ T cells (up to 1.5%).

### MVA-gBΔfur731 vaccination induces polyfunctional CD4^+^ T cell response

**Fig 4:**
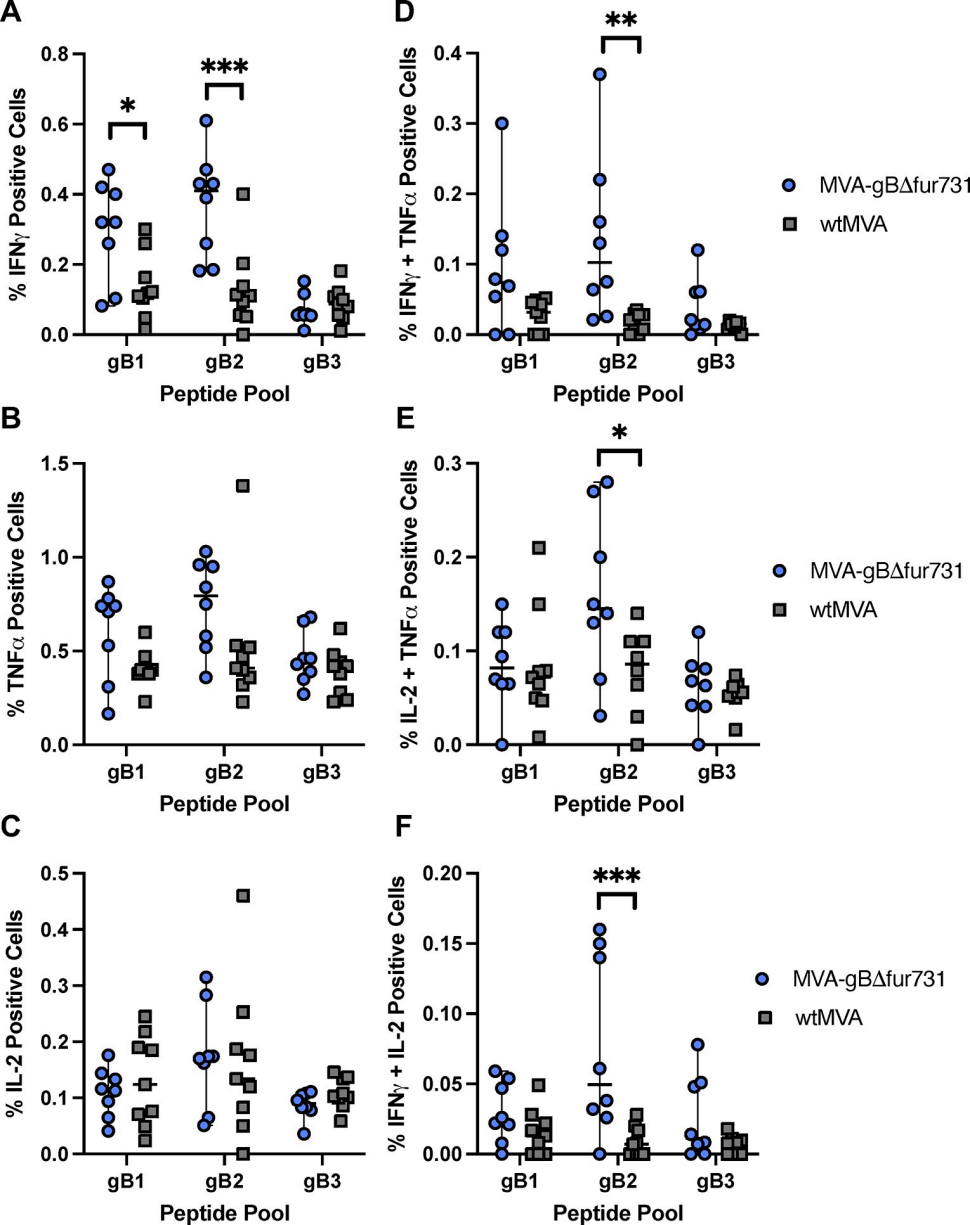
MVA-gBΔfur731 induces polyfunctional CD4^+^ T cell responses in immunized mice. Percentage of CD4^+^ T cells positive for single cytokine activation markers IFNγ (**A**), TNFα (**B**), or IL-2 (**C**), or double positive cytokine activation markers IFNγ/TNFα (**D**), IL-2/TNFα (**E**), or IFNγ/IL-2 (**F**). CD4^+^ T cells isolated from splenocytes of immunized mice were assessed for cytokine production after gB peptide pool stimulation. Blue circles represent splenic T cells from animals immunized with MVA-gBΔfur731 and gray squares represent mice immunized with wtMVA. Median values ± SD for each group are shown as horizontal lines and error bars. Asterisks (*/**/***) indicate statistically significant differences (*p*<0.05/*p*<0.01/*p*<0.001) between stimulation groups or MVA-gBΔfur731 and wtMVA immunized groups, as determined by two-way ANOVA with Tukey’s multiple comparisons test. A *p* value < 0.05 denotes significance.

While cytokine production is crucial for T cell activation and function, T cell polyfunctionality has also been associated with preventing other herpesvirus infections [59]. Therefore, after evaluating total cytokine producing T cells post-vaccination, we aimed to assess the polyfunctionality of cytokine producing T cells. We investigated specific cytokine producing CD4^+^ T cells populations in vaccinated mice splenocytes by measuring CD4^+^ cells producing individual cytokines or multiple cytokines. Mice vaccinated with MVA-gBΔfur731 induced significantly higher levels of CD4^+^/IFNγ^+^ cells when stimulated with peptide pools gB1 and gB2 as compared to wtMVA vaccinated mice (Fig 4A). While CD4^+^/TNFα^+^ cells were generally increased in MVA-gBΔfur731 vaccinated mice compared to those that received wtMVA, the trend was not statistically significant (Fig 4B). Conversely, MVA-gBΔfur731 and wtMVA vaccinated mice had similar levels of CD4^+^/IL-2^+^ splenocytes when stimulated with gB peptide pools (Fig 4C).

Although a smaller proportion of CD4^+^ T cells were double positive for cytokine markers, several populations were significantly increased in MVA-gBΔfur731 vaccinated mice over wtMVA vaccinated controls. CD4^+^ T cells double positive for IFNγ^+^/TNFα^+^ were significantly increased in MVA-gBΔfur731 vaccinated mice splenocytes when treated with the gB2 peptide pool (Fig 4D). Additionally, splenocytes from MVA-gBΔfur731 vaccinated mice had significantly higher populations of IL-2^+^/TNFα^+^ CD4^+^ T cells when stimulated with the gB2 peptide pool as compared to wtMVA vaccinated splenocytes (Fig 4E). Lastly, MVA-gBΔfur731 vaccinated mouse splenocytes stimulated with gB2 had a significantly greater proportion of IFNγ^+^/IL-2^+^ CD4^+^ T cells when compared to gB2 stimulated, wtMVA vaccinated splenocytes (Fig 4F). Taken together, these data suggest that the MVA-gBΔfur731 vaccine elicits strong polyfunctional CD4^+^ T cell responses.

### MVA-gBΔfur731 vaccination induces polyfunctional CD8^+^ T cell response

**Fig 5:**
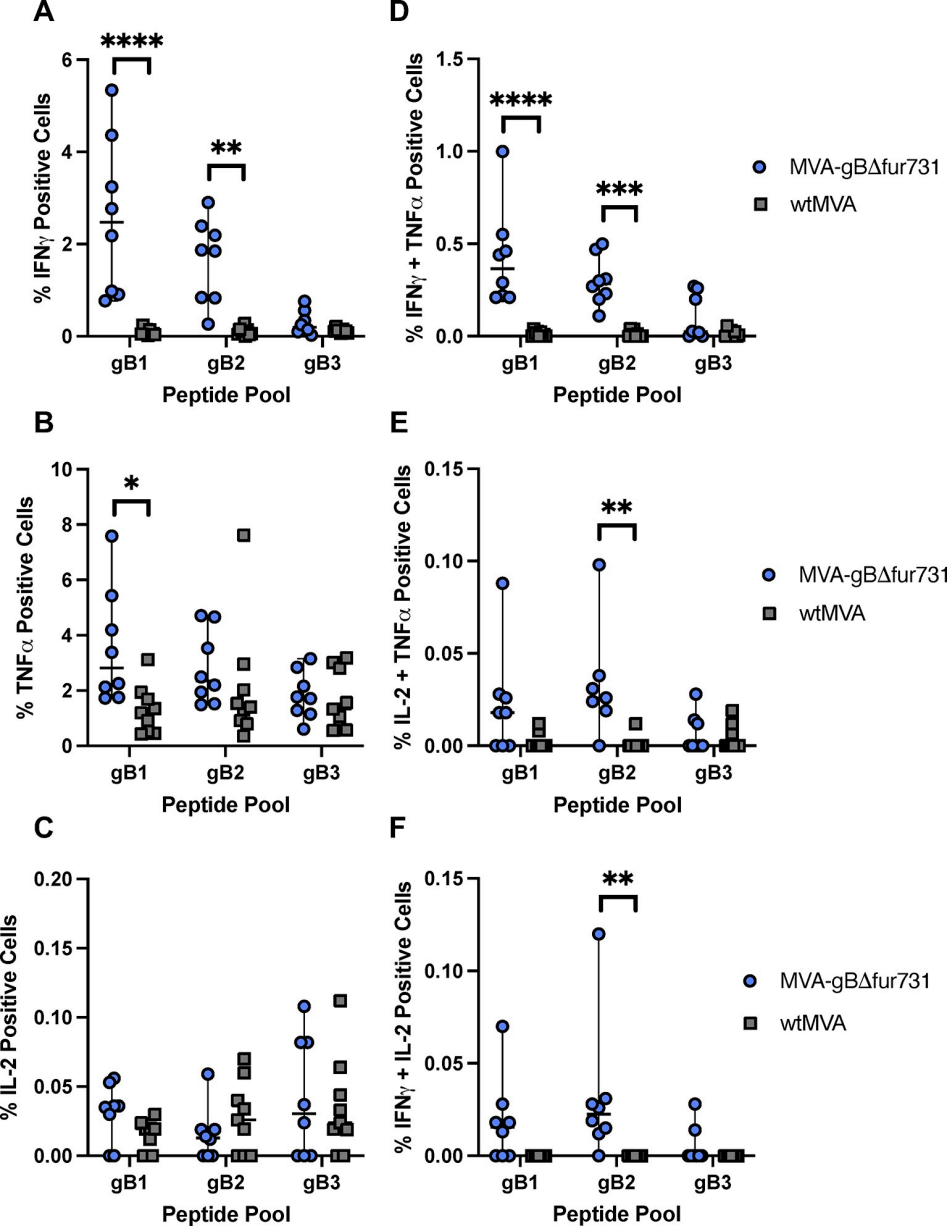
MVA-gBΔfur731 induces polyfunctional CD8^+^ T cell responses in immunized mice. CD8^+^ T cells positive for single cytokine activation markers IFNγ (**A**), TNFα (**B**), or IL-2 (**C**), or double positive cytokine activation markers IFNγ/TNFα (**D**), IL-2/TNFα (**E**), or IFNγ/IL-2 (**F**). CD8^+^ T cells isolated from splenocytes of immunized mice were assessed for cytokine production after gB peptide pool stimulation. Blue circles represent splenic T cells from animals immunized with MVA-gBΔfur731 and gray squares represent mice immunized with wtMVA. Median values ± SD values for each group are shown as horizontal lines and error bars. Asterisks (*/**/***/****) indicate statistically significant differences (*p*<0.05/*p*<0.01/*p*<0.001/*p*<0.0001) between stimulation groups or MVA-gBΔfur731 and wtMVA immunized groups, as determined by two-way ANOVA with Tukey’s multiple comparisons test. A *p* value < 0.05 denotes significance.

Due to the importance of CD8^+^ T cells in killing virally-infected cells, we sought to measure the polyfunctionality of the CD8^+^ T cell subset as well. Populations of CD8^+^/IFNγ^+^ cells were significantly increased in MVA-gBΔfur731 vaccinated splenocytes that had undergone gB1 or gB2 peptide stimulation, when compared to wtMVA vaccination (Fig 5A). Stimulation of MVA-gBΔfur731 vaccinated mouse splenocytes with the gB1 peptide pool also markedly increased CD8^+^/TNFα^+^ cells compared to those vaccinated with wtMVA (Fig 5B). Similar to CD4^+^ T cells, mice vaccinated with MVA-gBΔfur731 or wtMVA had comparable levels of CD8^+^/IL-2^+^ splenocytes regardless of gB peptide stimulation (Fig 5C).

Additionally, there were significant CD8^+^ T cell populations double positive for cytokine markers. As seen with CD8^+^/IFNγ^+^ cells, mice vaccinated with MVA-gBΔfur731 had significantly higher proportions of CD8^+^ cells double positive for IFNγ^+^/TNFα^+^ when treated with gB1 or gB2 peptides, as compared to those that received the wtMVA vaccine (Fig 5D). Finally, MVA-gBΔfur731 vaccinated mouse splenocytes stimulated with the gB2 peptide pool had significantly greater proportions of IL-2^+^/TNFα^+^ and IFNγ^+^/IL-2^+^ CD8^+^ T cells when compared to wtMVA vaccinated mice (Fig 5E, 5F). Overall, these results indicate that the MVA-gBΔfur731 vaccine induces vigorous and specific polyfunctional CD8^+^ T cell activation.

## Discussion

EEHV can cause lethal disease in Asian elephants throughout the world and is the leading cause of death in juvenile elephants living in North America and Europe. Despite the availability of sensitive tests and improved protocols for treating EEHV-associated illness, many recent cases have not had a positive outcome. An effective vaccine will contribute significantly towards conservation efforts for this endangered species. As a first and critical step in this endeavor, we generated a recombinant MVA that expresses the EEHV1A gB surface glycoprotein, which is a known immunodominant target for CMI and elicits significant antibody responses in elephants [16, 35, 60]. Mice vaccinated with this recombinant virus generated robust antibody and polyfunctional CD4^+^ and CD8^+^ T cell responses against EEHV1A gB. No mortality or abnormal clinical signs were observed in vaccinated mice during the course of the study. Therefore, these data support the potential use of this vaccine in elephants.

MVA is a novel and versatile platform for vaccine development against infections for a number of pathogens. While MVA has been used to develop a vaccine against some herpesviruses [61–63], and especially hCMV [29, 44, 64–66], its use in EEHV vaccinology has not yet been explored. One major advantage of using MVA for vaccine development is the ability to incorporate multiple target antigens into a single MVA vector. While the gB glycoprotein has demonstrated robust initial immunogenicity in our mouse model, we postulate that additional vaccine target antigens may be required in conjunction with gB to elicit immunity required to protect Asian elephants against severe or lethal disease. Previous studies by our group have identified other immunogenic EEHV antigens, including glycoproteins H and L, and the putative regulatory protein E40 [35]. Future studies will incorporate gH, gL, and E40 antigens into the MVA-gBΔfur731 vaccine construct and these vaccines will be tested *in vivo*. Another benefit of using MVA as a viral vectored vaccine is its inability to replicate in most mammalian cells [57, 58]. While elephant cells appear to be somewhat more permissive for MVA than 293T cells (Fig 1), we observed that there was no substantial increase in MVA-gBΔfur731 titers in EECs over a 72-hour period. These data indicate that EECs are still nonpermissive for MVA, which is a safety feature of our EEHV vaccine.

Significant gB-specific antibody titers were generated from all three groups (prime, prime-boost, and prime-2 boosts) that received the MVA-gBΔfur731 vaccination. These data indicate our MVA-gBΔfur731 vaccine candidate induces robust humoral immune responses, regardless of follow-up vaccine boosters. While these data indicate one or two vaccines will be enough to elicit EEHV antibody responses in vulnerable Asian elephant populations, additional long-term immune response studies will be required to determine the durability of these responses. Furthermore, a limitation of our model is the inability to measure neutralizing EEHV antibody titers post-vaccination. This limitation stems from the inability to culture EEHV *in vitro*, making it difficult to develop neutralization assays and measure neutralizing antibodies. Future studies will focus on generating elephant cell lines or organoids for the growth and characterization of EEHV, as well as developing assays to measure neutralizing antibody titers. These assays for evaluation of EEHV immunity may be particularly important since no challenge model currently exists for EEHV.

One potential limitation of this vaccine prototype is that it most likely expresses a post-fusion form of gB. However, prior studies with a similar MVA recombinant virus expressing CMV gB indicate that neutralizing antibodies were induced despite this potential limitation [52–54]. To our knowledge, other herpesvirus gB-based vaccines, which presumably express post-fusion forms of the proteins, have also not led to antibody-dependent enhancement (ADE) of disease. In addition, it is not likely that the post-fusion form of EEHV gB will have a significant effect on induction of effective cell-mediated responses mediated by T cells. Furthermore, previous studies also found that prior immunity against Vaccinia virus did not impair the ability of the recombinant MVA to stimulate anti-gB immune responses, indicating that booster vaccinations with MVA-based vaccines may still be effective [52].

Our EEHV1A MVA-gBΔfur731 vaccine elicited robust CD4^+^ and CD8^+^ T cell responses *in vivo*. These data are consistent with previous reports citing induction of both CD4^+^ and CD8^+^ T cell responses against gB during other herpesvirus infections [17, 67–71]. While CD8^+^ T cells are predominately responsible for cytolytic killing of virally-infected cells, CD4^+^ T cells are also known to activate CD8^+^ T cells by inducing cellular migration [72]. More recent studies also indicate that CD4^+^ T cells may possess cytolytic activity as well [69, 73–75]. Therefore, activation of both CD4^+^ and CD8^+^ T cell populations may be important for stimulating the highest level of immune protection. Additionally, MVA-gBΔfur731 induced higher amounts of polyfunctional CD8-specific T cell responses. Similarly, the related herpesvirus hCMV, has also been seen to induce high CD8^+^ T cell responses during infection [76]. While this study demonstrated effective T_H_1 (anti-viral) T cell activation, future studies will incorporate additional cellular cytokine markers to ensure a T_H_2 (anti-parasitic) cytokine response is not being induced by our MVA-gBΔfur731 vaccine. Additionally, while splenocytes were obtained from prime or singly boosted mice, no significant cellular responses were detected from these mice. Because we used splenocytes that were previously frozen, these results may be due poor viability of the cells, which dampened the sensitivity of our assays. Future studies will focus on repeating results using freshly harvested splenocytes.

The most vigorous T cell responses were induced by peptide pools representing amino acid residues 1–280 (gB1) and 281–560 (gB2). Whether these responses are driven by a few immunodominant epitopes or the sum of many is unknown and is determined by the intrinsic characteristics of some of the peptides/epitopes derived from gB, from the presence in the host of appropriate MHC molecules and T cell receptors, and events related to antigen processing and presentation. In our previous study in chronically infected adult elephants, responses were detected to all three gB peptide pools, although the amplitude of responses varied for each pool depending on the individual [35]. The landscape of class I and class II MHC molecules in elephants is unknown and how that compares to the outbred mice used in this current study is correspondingly unknown. Therefore, it remains to be seen if elephant T cell responses will share similar immunodominant epitopes as those observed in mice following vaccination with MVA-gBΔfur731.

Although MVA is an appropriate platform for vaccine development, many studies have shown that heterologous prime-boost regimens can be advantageous for protection against viral infection [77]. Future directions will assess the use of a subunit vaccine platform in conjunction with the MVA-gBΔfur731 vaccine described here to determine whether this approach will elicit more robust and durable immune responses than either platform alone.

### Conclusion

Elephant endotheliotropic herpesvirus (EEHV) infection is the leading cause of mortality in juvenile Asian elephants under human care in North America and Europe and is now recognized as a significant cause of death in both captive and wild range country juvenile Asian elephants. Hence, an effective vaccine with the ability to prevent mortality caused by EEHV would significantly improve the conservation efforts for endangered Asian elephants. Our findings demonstrate that an MVA-based EEHV vaccine expressing the extracellular domain of EEHV1A gB induces robust immunity in an animal model and supports its potential use in elephants.

## Supporting information

S1 Raw image (PDF)

S2 Raw image (PDF)
