# Supplementary figures and images for: Modified vaccinia Ankara expressing EEHV1A glycoprotein B elicits humoral and cell-mediated immune responses in mice
Source: PLoS One. 2022 Mar 21;17(3):e0265424. doi: 10.1371/journal.pone.0265424 (PMC8936464; doi:10.1371/journal.pone.0265424)

| Un | wt  | MVA |
|----|-----|-----|
|    | MVA | -gB |

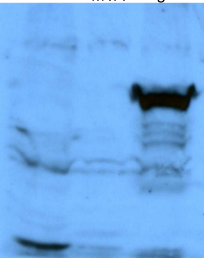

Supplement: S1 Raw image — (PDF) [file pone.0265424.s001.pdf]

Un

MVA

MVA  
-gB

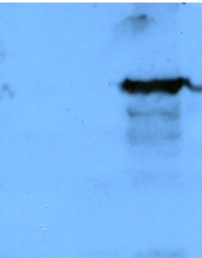

Supplement: S2 Raw image — (PDF) [file pone.0265424.s002.pdf]
